# The disordered negatively charged *C*‐terminus of the large HECT E3 ubiquitin ligase HERC2 provides structural and thermal stability to the HECT *C*‐lobe

**DOI:** 10.1002/pro.5229

**Published:** 2024-11-20

**Authors:** Kelly L. Waters, Kayla J. Rich, Noah D. Schwaegerle, Tianyi Yang, Shuanghong Huo, Donald E. Spratt

**Affiliations:** ^1^ Gustaf H. Carlson School of Chemistry and Biochemistry Clark University Worcester Massachusetts USA

**Keywords:** AlphaFold, circular dichroism, disordered tail, DNA damage response, HECT E3 ubiquitin ligase, HERC2, NMR spectroscopy, protein recruitment, ubiquitin

## Abstract

Homologous to the *C*‐terminus of E6AP (HECT) and RCC1‐like domain (RLD)‐containing protein 2 (HERC2) is a large, 528 kDa E3 ubiquitin ligase that is associated with cancer, oculocutaneous albanism type 2, Prader‐Willi syndrome, and other neurological diseases. HERC2 has been found to contribute to double‐stranded DNA break repairs, tumor suppression, maintaining centrosome architecture, and ubiquitylation. The *C*‐terminal portion of the HECT domain (*C*‐lobe) of HERC2 is responsible for transferring ubiquitin to a substrate but the precise function of the other eight domains in HERC2 are unknown. Interestingly, HERC2 contains a unique and negatively charged *C*‐terminal tail adjoined to the *C*‐lobe that is predicted to act as a linker to promote interactions between HERC2 and its binding partners. This study aims to better understand the function and relevance of HERC2 in disease by investigating the structural aspects of the HERC2 *C*‐lobe and HERC2 *C*‐terminal tail using AlphaFold followed by molecular dynamics (MD) simulations, multidimensional nuclear magnetic resonance (NMR), and circular dichroism (CD). Secondary structure content analysis from MD simulations and the fully resonance assigned ^1^H‐^15^N HSQC spectra of the HERC2 *C*‐lobe and the isolated *C*‐terminal tail confirm that the *C*‐lobe is well‐folded but the *C*‐terminal tail is disordered. CD melting curves indicate that the flexible *C*‐terminal tail provides improved stability to the *C*‐lobe. Additionally, MD simulations have identified that the interaction between residues D4829 and R4728 is prevalent among the non‐bonded contacts between the tail and the *C*‐lobe. Overall, our results demonstrate that the negatively charged *C*‐terminal tail is disordered, provides stability to the *C*‐lobe, and may act as a flexible scaffold for protein–protein interactions.

## 1 INTRODUCTION

Ubiquitylation is a post‐translational modification (PTM) that regulates various biological processes by covalently attaching a small 8 kDa protein, ubiquitin (Ub), onto a substrate (Hershko and Ciechanover 1998). Ubiquitylation is carried out by a three‐enzyme cascade that involves an E1‐activating enzyme (E1), E2‐conjugatiing enzyme (E2), and an E3 ubiquitin ligase (E3). The E1 catalyzes an ATP‐dependent reaction resulting in an “activated” high energy thioester bond between the E1 catalytic cysteine and ubiquitin *C*‐terminal glycine (E1 ~ Ub) (Hershko and Ciechanover 1998; Komander and Rape 2012). The ubiquitin is transferred via a transthiolation reaction from the E1 ~ Ub to the E2 catalytic cysteine (E2 ~ Ub) (Hershko and Ciechanover 1998; Komander and Rape 2012). An E3 ubiquitin ligase is then recruited to the E2 ~ Ub complex and promotes the attachment of ubiquitin to the substrate by a mechanism unique to the type of E3 ubiquitin ligase recruited (Komander and Rape 2012).

The homologous to E6AP *C*‐terminus (HECT) E3 ligases use their HECT domain, consisting of a bi‐lobal structure (i.e., *N*‐lobe and *C*‐lobe), to transfer ubiquitin from an E2 ~ Ub complex. In this mechanism, the HECT *N*‐lobe recognizes the E2 ~ Ub complex and promotes transthiolation of ubiquittin from the E2 ~ Ub complex to the conserved catalytic cysteine located in the HECT *C*‐lobe (E3 ~ Ub) (Huang et al. 1999). The ubiquitin‐charged HECT domain then recognizes a target protein and catalyzes the transfers of the ubiquitin cargo onto the target protein, forming a stable isopeptide between the ubiquitin *C*‐terminal glycine and the ε‐amine of a substrate lysine (Huang et al. 1999; Scheffner et al. 1995). The ubiquitylation of an intracellular target protein has been shown to regulate a myriad of biological functions including proteasomal degradation, DNA damage recognition, apoptosis, cell division, and transcriptional regulation (Komander and Rape 2012). Each HECT E3 ligase carries out a unique set of functions via their ubiquitylation activity and ultimately decides the fate of the ubiquitin‐tagged protein (Wang et al. 2020). There are many HECT E3 ligases whose function is yet to be fully understood, with HECT and RCC1‐like domain (RLD)‐containing protein 2 (HERC2) among these.

HERC2 is a massive 527 kDa E3 ubiquitin ligase that has been implicated in ulcerative colitis, cancer, Prader‐Willi syndrome, Angelman syndrome, and other neurological diseases (Bekker‐Jensen et al. 2010; Bonanno et al. 2016; Cubillos‐Rojas et al. 2017; Franke et al. 2008; Harlalka et al. 2013; Ibarrola‐Villava et al. 2010; Imai et al. 2015; Izawa et al. 2011; Ji et al. 1999; Liu et al. 2023; Puffenberger et al. 2012). Recent proteomic studies have shown that HERC2 interacts with many proteins involved in the DNA damage response, tumor suppression, centrosome architecture, intracellular protein transport/trafficking, translation, iron metabolism, and proteasomal degradation via ubiquitylation (Galligan et al. 2015).

**FIGURE 1:**
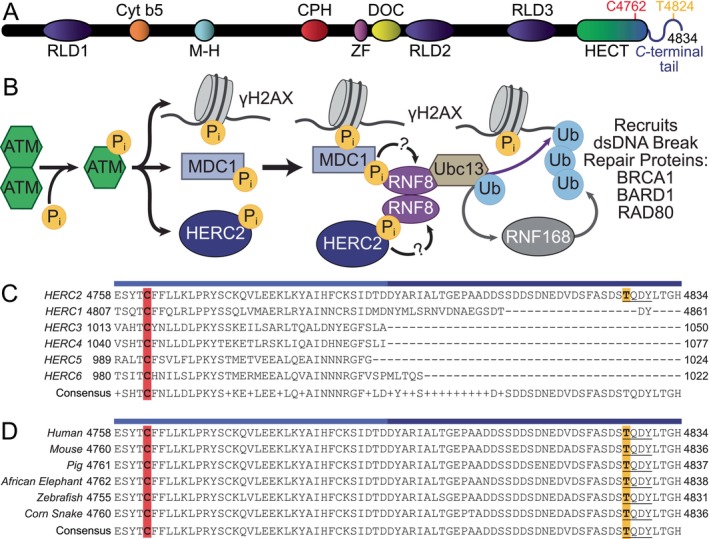
HERC2 domain architecture, multiple sequence alignments of the HERC2 *C*‐terminal tail, and the potential role of HERC2 in double strand DNA break repair. (a) HERC2 is 4834 amino acids long and contains nine domains: RLD1 (purple; residues 415–778), Cyt‐b5 (orange; residues 1207–1283), M‐H (cyan; residues 1859–1932), CPH (red; residues 2554–2631), ZF (pink; residues 2702–2755), DOC (yellow; residues 2774–2914), RLD2 (purple; residues 2958–3326), RLD3 (purple; residues 3951–4318), and HECT (green and blue; residues 4457–4794). The HECT domain is comprised of a bi‐lobal structure with an *N*‐lobe (green; residues 4457–4675) and a *C*‐lobe (blue; residues 4676–4794) which contains the catalytic cysteine (red; residue C4762). HERC2 also has a negatively charged *C*‐terminal tail (navy blue; residues 4793–4834) that contains the threonine (purple; residue T4827) thought to be involved in protein recruitment during the DNA damage response. (b) When a dsDNA break is detected, kinase ATM is activated such that it will recognize TQ or SQ motifs and phosphorylate residue T4827 of HERC2. RNF8 then binds to phosphorylated HERC2, allowing for the subsequent recruitment of other double strand DNA break repair proteins to the damaged DNA site. (c) The *C*‐terminal end of HERC2 (residues 4758–4834; UniProt: O95714) is unique when aligned to the *C*‐terminal regions of the other HERC subfamily members: HERC1 (residues 4807–4861; UniProt: Q15751), HERC3 (residues 1013–1050; UniProt: Q15034), HERC4 (residues 1040–1057; UniProt: Q5GLZ8), HERC5 (residues 989–1024; UniProt: Q9UII4), and HERC6 (residues 980–1022; UniProt: Q8IVU3). (d) The *C*‐terminal end of HERC2 was found to be well conserved among higher order eukaryotes: Human (residues 4758–4834; UniProt: O95714), mouse (residues 4760–4836; UniProt: Q4U2R1), pig (residues 4761–4837; UniProt: E1B782), African elephant (residues 4762–4838; UniProt: G3STX9), Zebrafish (residues 4755–4831; UniProt: A0A8M9QG78), and corn snake (residues 4760–4836; UniProt: A0A6P9D527). The “TQDY” ATM recognition motif (underlined) is present in all species. The catalytic cysteine of each HECT is highlighted red and the threonine thought to be involved in protein recruitment during the dsDNA break response is highlighted yellow. All sequences were aligned using Clustal Omega (https://www.ebi.ac.uk/ebisearch) and visualized in Jalview (Madeira et al. 2022; Waterhouse et al. 2009).

The ability for HERC2 to contribute to these diverse set of functions and protein–protein interactions have been attributed to its nine unique domains (Figure 1a). These include three RLD domains (RLD1, residues P415–S778; RLD2, residues R2958–T3326; and RLD3, residues S3951–T4318). RLD domains have been found to carry out various functions involved in the cell cycle, vesicular trafficking, guanine nucleotide exchange, and more (Hadjebi et al. 2008). While the functions of RLD1 and RLD3 remain unknown, the RLD2 of HERC2 has been observed to regulate E6AP autoubiquitylation, another HECT E3 ubiquitin ligase whose malfunction is connected to the neurological disease, Angelman syndrome (Kishino et al. 1997; Kühnle et al. 2011). HERC2 also contains a non‐heme‐binding cyt‐b5 like domain (cyt‐b5, residues V1207–L1283), a Mind‐bomb‐HERC2 domain (M‐H, residues S1859–P1932), and a downregulated in ovarian cancer domain (DOC, residues Q2774–R2914), whose roles and functions remain unclear. The Cullin7‐PARC‐HERC2 domain (CPH, residues R2554–P2631) is predicted to bind to tetrameric p53 and regulate p53 activity (Cubillos‐Rojas et al. 2014). The ZZ‐type Zinc finger domain (ZF, residues I2702–Q2755) has been shown to bind to histone H3 and Small ubiquitin‐like modifier 1 (SUMO1), thus confirming the colocalization of HERC2 to DNA (Danielsen et al. 2012; Liu et al. 2020). The HERC2 HECT domain (residues D4457–D4794; *N*‐lobe, residues D4457–C4675; *C*‐lobe, residues G4676–D4794) contains the catalytic cysteine (C4762) is responsible for HERC2‐dependent ubiquitylation. Adjoining to the HERC2 HECT domain is a unique negatively charged *C*‐terminal tail (residues T4793–H4834).

The HERC2 negatively charged *C*‐terminal tail is theorized to be phosphorylated at T4827 and may contribute to protein recruitment during the double‐stranded DNA break response (Bekker‐Jensen et al. 2010; Huen et al. 2007). In response to double strand DNA breaks, ataxia‐telangiectasia mutated kinase (ATM) recognizes SQ or TQ motifs and phosphorylates T4827 of HERC2 to promote a series of interactions (Bekker‐Jensen et al. 2010; Matsuoka et al. 2007; Paull 2015; Stucki et al. 2005). Another E3 ubiquitin ligase, RING finger protein 8 (RNF8), is theorized to interact with HERC2 using its forkhead‐associated (FHA) domain, which selectively binds to phosphothreonines with a phenylalanine or tyrosine in the i + 3 position (i.e., TQDY, HERC2 residues T4827–Y4830) (Bekker‐Jensen et al. 2010; Huen et al. 2007; Kolas et al. 2007). This HERC2–RNF8 interaction is thought to lead to the recruitment of the E2 conjugating enzyme, UBE2N (aka Ubc13), which promotes histone ubiquitylation and recruits other dsDNA break repair proteins including RING finger protein 168 (RNF168), Breast cancer type 1 susceptibility protein (BRCA1), BRCA1‐associated RING domain protein 1 (BARD1), and receptor‐associated protein 80 (RAP80) (Figure 1b) (Becker et al. 2021; Chen and Sleckman 2022; Dai et al. 2021; Huen et al. 2007; Krais et al. 2021; Ma et al. 2011; Mailand et al. 2007; Mattiroli et al. 2012; Orr and Savage 2015; Thorslund et al. 2015; Walsh 2015; Witus et al. 2021). While RNF8 and HERC2 have been found to interact via pulldown assays with whole cell extracts, this interaction has yet to be fully characterized and the role of HERC2 *C*‐terminal tail remains unclear (Bekker‐Jensen et al. 2010).

Interestingly, multiple sequence alignments (MSAs) using Clustal Omega (https://www.ebi.ac.uk/ebisearch) indicate that the negatively charged *C*‐terminal tail is a unique feature of HERC2 that shows high conservation across eukaryotes (Madeira et al. 2022). In an MSA of the *C*‐terminal regions of all HERC‐type HECT E3 ubiquitin ligases, HERC2 is the only HECT to contain a negatively charged and elongated tail after the *C*‐lobe (Figure 1c). This region of HERC2 shows high conservation among various species (Figure 1d). Interestingly, the ATM and the E3 ubiquitin ligase RING finger protein 8 (RNF8) recognition motif (i.e., TQDY, residues T4827–Y4830) is included in this region.

Despite being a unique and conserved feature of HERC2, the structural aspects of the negatively charged *C*‐terminal tail have yet to be fully investigated. Using computational molecular models, multidimensional nuclear magnetic resonance (NMR), and circular dichroism, this study aims to understand the structural aspects of the HERC2 negatively charged *C*‐terminal tail and the adjacent HECT *C*‐lobe.

## 2 RESULTS

### 2.1 Initial secondary structure analysis and identification of potential protein binding sites

The online servers PSIPRED (McGuffin et al. 2000) and DISOPRED3 (Jones and Cozzetto 2015) were used to predict the secondary structure, intrinsically disordered regions, and potential binding sites of HERC2 extended *C*‐lobe (residues G4676–H4834). PSIPRED predicted there to be one potential α‐helical region (residues Y4796–L4801) in the HERC2 negatively charged *C*‐terminal tail with a high confidence (>5). DISOPRED3 predicted that the HERC2 negatively charged *C*‐terminal tail (residues G4803–H4834) is intrinsically disordered and may contain potential protein binding sites with high confidence values (>0.9) in the following regions: S4811, S4814‐F4822, and Q4828‐H4834. These initial results support the hypothesis that the HERC2 *C*‐terminal tail is disordered and contains various potential binding sites which may help mediate protein–protein interactions such as the HERC2 (residues T4827–Y4830) and RNF8 FHA interaction (Bekker‐Jensen et al. 2010). To verify these initial predictions and further understand the structural aspects of the HERC2 negatively charged *C*‐terminal tail, the HERC2 constructs (extended *C*‐lobe, *C*‐lobe, and negatively charged *C*‐terminal tail) were modeled using AlphaFold followed by molecular dynamics (MD) simulations and investigated using heteronuclear multidimensional NMR spectroscopy.

### 2.2 AlphaFold modeling of the HERC2 *C*‐lobe constructs

**FIGURE 2:**
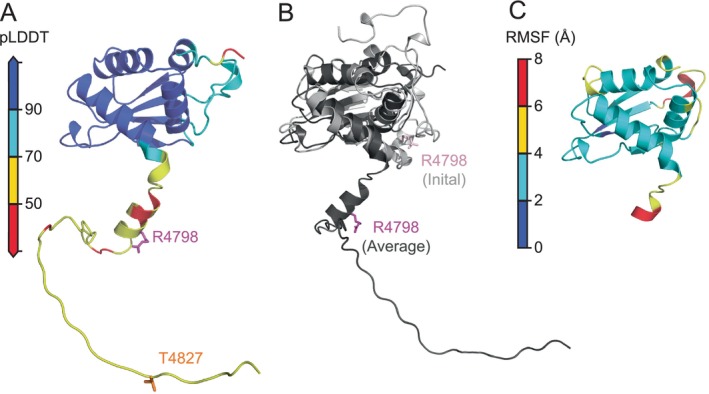
AlphaFold predicted structures of HERC2 *C*‐lobe (residues P4681–A4797) and HERC2 extended *C*‐lobe (residues G4676–H4834). (a) The AlphaFold predicted structure of HERC2 extended *C*‐lobe (residues G4676–A4797) was mapped with the pLDDT values based on the confidence level: <50 (red), 50–70 (yellow), 70–90 (cyan), >90 (dark blue). The first residue of the *C*‐terminal tail (R4798 in magenta) and the site of ATM‐mediated phosphorylation (T4827 in orange) are shown as sticks. Overall, the *C*‐lobe is predicted with high confidence as shown in blue but contains a loop region (residues V4723–R4739) located between α‐helix 3 and β‐strand 2 with lower confidence as shown in cyan. The negatively charged *C*‐terminal tail region has a much lower confidence as indicated in red and yellow. (b) The predicted superposition of the initial (light gray) and average (dark gray) HERC2 extended *C*‐lobe structure (residues G4676–H4834) is shown. The position of residue. R4798 is shown in light pink (initial) and magenta (average). (c) The AlphaFold predicted 3D structure of HERC2 *C*‐lobe (P4681‐A4797) is mapped with the values of Cα root mean squared fluctuation (RMSF) based on the range of fluctuation: 0–2 Å (dark blue), 2–4 Å (cyan), 4–6 Å (yellow), and 6–8 Å (red). The most fluctuation is shown in yellow and red at the *C*‐terminal α‐helix (residues D4794–A4797) and *C*‐lobe loop (residues R4726–R4739) between α‐helix 3 and β‐strand 2. This supports the conclusion that the structural integrity of the *C*‐lobe is not compromised in the presence of the HERC2 *C*‐terminal tail.

AlphaFold Colab was used to predict the 3D structures of the HERC2 *C*‐lobe (residues G4676–A4797) and HERC2 extended *C*‐lobe (residues G4676–H4834). The accuracy of the predicted extended *C*‐lobe structure was determined by calculating the per‐residue model confidence value (pLDDT) (Figure 2a). As expected, due to similar structures for other available in the PDB, most residues found in the HERC2 *C*‐lobe are modeled with high accuracy (pLDDT >90); however, the residues within the negatively charged *C*‐terminal tail were modeled with low accuracy (pLDDT <50). The low pLDDT, DISOPRED3 intrinsically disordered regions, and PSIPRED low confidence α‐helices predictions all imply that the negatively charged *C*‐terminal tail of HERC2 is flexible and disordered. To illustrate the conformational deviations among the HERC2 extended *C*‐lobe, the average simulated structure was superimposed over the initial structure of the HERC2 extended *C*‐lobe (Figure 2b). The significant overlap within most regions of the HERC2 *C*‐lobe indicates there is little structural deviation and the *C*‐lobe remains folded. However, the large deviations that are found within the *C*‐lobe loop (residues R4726–R4739 between α‐helix 3 and β‐strand 2) and the negatively charged *C*‐terminal tail suggest that the structure in these regions is more flexible and disordered.

To evaluate the structural integrity of the HERC2 *C*‐lobe in the presence of the HERC2 Tail, the Cα RMSD (root‐mean‐square deviation) were determined for the HERC2 *C*‐lobe (residues P4681–A4797) and the HERC2 extended *C*‐lobe (residues G4676–H4834). Overall, the RMSD are similar, but large fluctuations are found in the *C*‐terminal α‐helix (residues D4794–A4797) and *C*‐lobe loop (residues R4726–R4739) between α‐helix 3 and β‐strand 2 (Figure 2c). These computational data indicate that the *C*‐lobe maintains the same 3D structure in the presence and absence of the negatively charged *C*‐terminal tail of HERC2.

### 2.3 Secondary structure content of the HERC2 constructs

**FIGURE 3:**
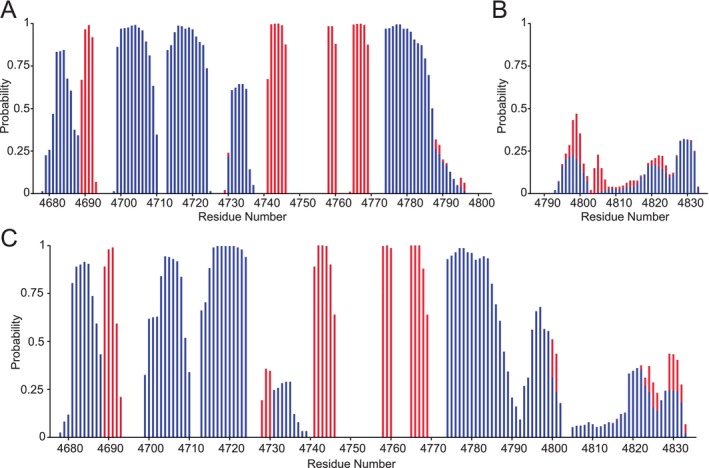
Secondary structure content analysis indicates that the *C*‐lobe is well folded and the negatively charged *C*‐terminal tail of HERC2 is disordered. (a) Secondary structure content analysis of the HERC2 *C*‐lobe (residues G4676–A4797) indicates that the *C*‐lobe is primarily folded and made up of α‐helices (blue) and β‐strands (red). (b) Secondary structure content analysis of the HERC2 negatively charged *C*‐terminal tail (residues T4793–H4834) indicates that the HERC2 tail is primarily unfolded. (c) The HERC2 extended *C*‐lobe (residues G4676–H4834) is primarily folded and made up of α‐helices (blue) and contains some β‐strand and random coil regions.

To quantify the predicted structural models, the secondary structure content was analyzed using the MD trajectories for the HERC2 *C*‐lobe (residues G4676–A4797), HERC2 negatively charged *C*‐terminal tail (residues T4793–H4834), and HERC2 extended *C*‐lobe (residues G4676–H4834) (Figure 3). The HERC2 *C*‐lobe secondary structure content analysis indicates that the overall structure of the *C*‐lobe is well‐folded and contains α‐helices and β‐strands (Figure 3a). In contrast, the HERC2 negatively charged *C*‐terminal tail is primarily disordered (Figure 3b). The secondary structure content analysis of the HERC2 extended *C*‐lobe confirmed that the HERC2 extended *C*‐lobe is primarily made of α‐helices and random coil in the tail region (Figure 3c). When the HERC2 *C*‐lobe and HERC2 extended *C*‐lobe secondary structures are compared, the residues G4725–D4740 showed a decrease in predicted α‐helical content when the negatively charged *C*‐terminal tail was included. This suggests that this region may be involved in maintaining the structural integrity of the HERC2 extended *C*‐lobe when the negatively charged *C*‐terminal tail is present.

### 2.4 NMR spectra support the secondary structure content analysis from MD simulations

**FIGURE 4:**
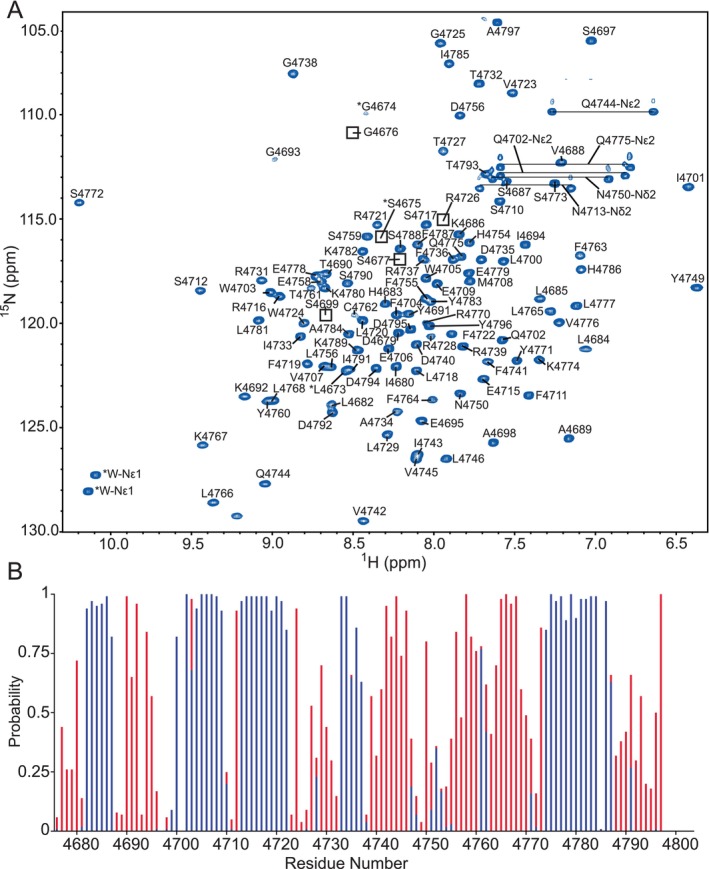
Assigned ^15^N‐HSQC spectrum and NMR‐based secondary structure analysis indicate that the HERC2 *C*‐lobe is well‐folded. (a) The spectrum of HERC2 *C*‐lobe (residues G4676–A4797) was assigned using NMRViewJ according to the single‐letter amino acid code and residue number. Artifact residues remaining after the PreScission protease cleavage of the *N*‐terminal GST‐fusion tag that appear in the spectrum are indicated by asterisk (*). The NMR sample contained 1.5 mM of ^13^C/^15^N‐isotopically labeled HERC2 *C*‐lobe (residues G4676–A4797) in 20 mM Na_2_HPO_4_ (pH 7.0), 100 mM NaCl, 1 mM EDTA, 2 mM TCEP, and 10% D_2_O. All data were collected on a Varian Inova 600‐MHz NMR spectrometer. (b) Chemical Shift Index (CSI) NMR‐based secondary structure analysis indicates that the HERC2 *C*‐lobe is well folded and made up of 4 α‐helices (blue) and 4 β‐strands (red).

The ^15^N‐HSQC spectra for ^15^N/^13^C‐labeled HERC2 *C*‐lobe (residues G4676–A4797) and HERC2 negatively charged *C*‐terminal tail (residues T4793–H4834) were assigned using the standard biomolecular NMR backbone experiments (i.e., 3D HNCO, 3D HN(CA)CO, 3D HN(CO)CA, 3D HNCA, 3D HNCACB, and 3D CBCA(CO)NH). The assigned HERC2 *C*‐lobe ^15^N‐HSQC spectrum showed well‐dispersed amide peaks, which indicated that the *C*‐lobe is well folded (Figure 4a). The secondary structure content of the HERC2 *C*‐lobe was determined by inputting the backbone resonance assignments into the chemical shift index (CSI) web server from the Wishart Lab (http://csi3.wishartlab.com/cgi-bin/index.php). The CSI web server predicted that the HERC2 *C*‐lobe contains 4 α‐helicies, 4 β‐strands, and some disordered regions (Figure 4b).

**FIGURE 5:**
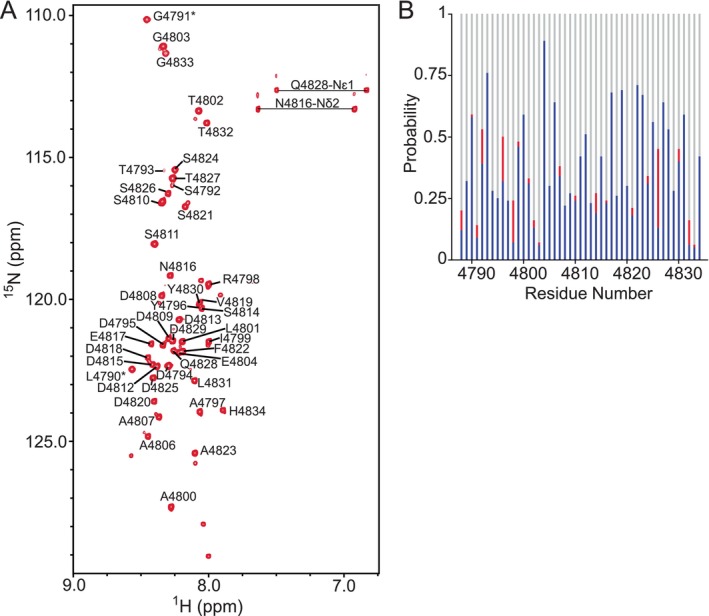
Assigned ^15^N‐HSQC spectrum and NMR‐based secondary structure analysis of indicate that the negatively charged *C*‐terminal tail of HERC2 is disordered. (a) The spectrum of HERC2 negatively charged *C*‐terminal tail (residues T4793–H4834) was assigned using NMRViewJ according to the single‐letter amino acid code and residue number. Artifact residues remaining after the PreScission protease cleavage of the *N*‐terminal GST‐fusion tag that appear in the spectrum are indicated by asterisk (*). The NMR sample contained 1.5 mM ^13^C/^15^N‐isotopically labeled HERC2 negatively charged *C*‐terminal tail (residues T4793–H4834) in 20 mM Na_2_HPO_4_ (pH 7.0), 100 mM NaCl, 1 mM EDTA, 2 mM TCEP, and 10% D_2_O. All data were collected on a Varian Inova 600‐MHz NMR spectrometer. (b) Chemical Shift Index (CSI) NMR‐based secondary structure indicates that the negatively charged *C*‐terminal tail of HERC2 (residues T4793–H4834) is predominantly random coil (gray) with lower probabilities for the presence of α‐helices (blue) and β‐strands (red).

In contrast, the ^15^N‐HSQC spectrum for the HERC2 negatively charged *C‐*terminal tail showed that this region is disordered as indicated by the narrow distribution of amide peaks (Figure 5a). CSI also predicted that the HERC2 negatively charged *C‐*terminal tail is primarily disordered (Figure 5b). Overall, the structural data for the HERC2 *C*‐lobe and HERC2 *C*‐terminal tail agree with the secondary structure content analysis from MD simulations, supporting the idea that the isolated HERC2 *C*‐lobe is well‐folded and isolated HERC2 *C*‐terminal tail is disordered.

**FIGURE 6:**
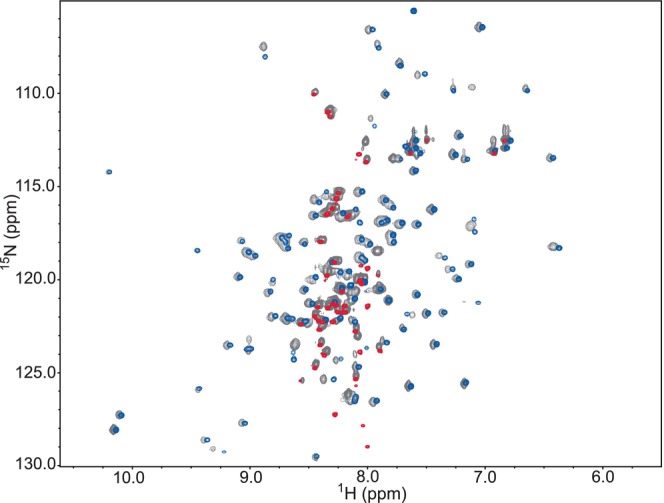
^15^N‐HSQC spectral overlay of the intact HERC2 extended *C*‐lobe construct with the isolated HERC2 *C*‐lobe and HERC2 negatively charged *C*‐terminal tail show significant spectral overlap. The HERC2 extended *C*‐lobe (gray; residues G4676–H4834), *C*‐lobe (blue; residues G4676–A4797), and negatively charged *C*‐terminal tail (red; residues T4793–H4834) were overlaid and found to have significant peak overlap, verifying that the isolated HERC2 *C*‐lobe and negatively charged *C*‐terminal tail are representative of their biological structures in solution.

With the HERC2 *C*‐lobe and the negatively charged *C‐*terminus being adjoined, it was necessary to determine if the solved HERC2 *C*‐lobe and HERC2 *C*‐terminal tail ^15^N‐HSQC spectra were similar to their native states when attached. To determine their biological relevance, a ^15^N‐HSQC spectrum was collected for the HERC2 extended *C*‐lobe (residues G4676–H4834). When this spectrum was overlaid with the ^15^N‐HSQC spectra for the isolated HERC2 *C*‐lobe and negatively charged *C*‐terminal tail, there was a significant amount of peak overlap (Figure 6). These data show that the isolated HERC2 *C*‐lobe and negatively charged *C*‐terminal tail constructs are biologically relevant since the chemical environment of most residues was not significantly different when compared the HERC2 extended *C*‐lobe spectrum. This suggests that the isolated HERC2 *C*‐lobe and negatively charged *C*‐terminal tail NMR spectra and secondary structure predictions are representative of the structure found in the intact HERC2 extended *C*‐lobe construct. Taken together, these data agree with the secondary structure content analysis from MD simulations and demonstrates that the HERC2 *C*‐lobe is well‐folded and primarily made up of α‐helices and β‐strands as seen in other HECT E3 ubiquitin ligase family members, but the unique negatively charged *C*‐terminal tail of HERC2 is disordered and flexible.

### 2.5 Investigating the effect of the negatively charged *C*‐terminal tail on HERC2 *C*‐lobe stability

**FIGURE 7:**
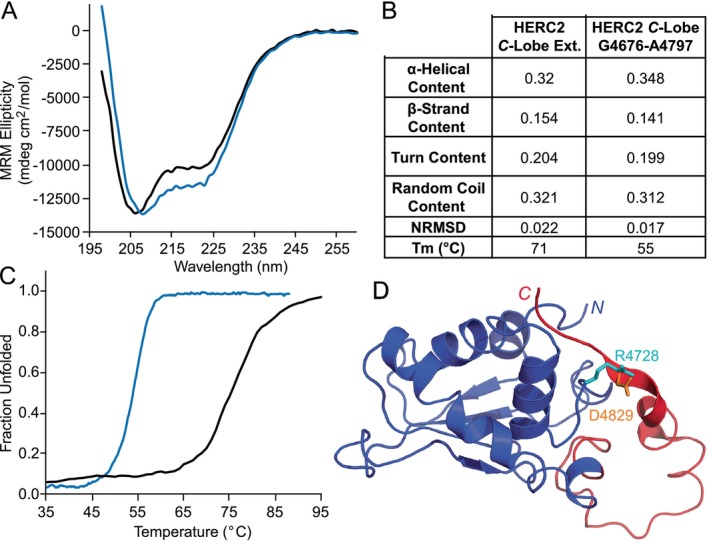
Circular dichroism (CD) spectra and normalized melting curves indicate that the HERC2 negatively charged *C*‐terminal tail increases the stability of the HERC2 *C*‐lobe. (a) The CD spectra of the HERC2 extended *C*‐lobe (residues G4676–H4834; in black) and the HERC2 *C*‐lobe (residues G4676–A4797; in blue) were collected from 260 to 200 nm. These spectra confirm that the secondary structure of the *C*‐lobe constructs is primarily α‐helical and that the HERC2 extended *C*‐lobe contains more random coil than the HERC2 *C*‐lobe alone. (b) The stability of the HERC2 *C*‐lobe constructs were measured at 222 nm while temperature was increased at 1°C/min. The extended *C*‐lobe and *C*‐lobe alone constructs were found to have melting temperatures of 71°C and 55°C, respectively. This indicates that the HERC2 extended *C*‐lobe shows more thermal stability than the HERC2 *C*‐lobe alone. (c) The secondary structure content calculated by CDPro is shown as fractions and the melting temperatures are shown in the table for the HERC2 constructs (Provencher and Glöckner 1981; Sreerama and Woody 2000). (d) Among the non‐bonded interactions between the *C*‐terminal tail and HERC2 *C*‐lobe, D4829–R4728 contact is predominant with a fraction of the population being greater than 0.5. R4728 (cyan) and D4829 (orange) are shown as sticks in the HERC2 extended *C*‐lobe structure such that the *C*‐lobe (G4676–A4797) and the negatively charged *C*‐terminal tail (R4798–H4834) are shown in blue and red, respectively.

The effect of the HERC2 negatively charged *C*‐terminal tail on the HERC2 *C*‐lobe stability was assessed using circular dichroism. The secondary structure content of the HERC2 extended *C*‐lobe and HERC2 *C*‐lobe was initially determined by measuring the molar ellipticity between 260 and 195 nm (Figure 7a). Visually, the spectra indicate that the secondary structures are primarily α‐helical with some random coil. Using the software CDPro and the CONTINLL method, HERC2 extended *C*‐lobe was calculated to be 32.0% α‐helical, 15.4% β‐strands, 20.4% turn, and 32.1% random coil (Figure 7b) (Provencher and Glöckner 1981; Sreerama and Woody 2000). The HERC2 *C*‐lobe construct is also calculated to be 34.8% α‐helical, 14.1% β‐strands, 19.9% turn, and 31.2% random coil (Figure 7b) (Provencher and Glöckner 1981; Sreerama and Woody 2000).

The thermal stability of the HERC2 *C*‐lobe and HERC2 extended *C*‐lobe were determined by measuring the molar ellipticity at the wavelength 220 nm while increasing the temperature. The melting curves illustrate that the HERC2 *C*‐lobe has a lower melting temperature of 55°C than the HERC2 extended *C*‐lobe which has a melting temperature of 71°C (Figure 7c). This indicates that the HERC2 negatively charged *C*‐terminal tail provides structural and/or thermal stability to the HERC2 *C*‐lobe. To investigate how the negatively charged *C*‐terminal tail might provide stability, we analyzed the non‐bonded contacts between the HERC2 tail and HERC2 *C*‐lobe throughout the stimulations. Two residues are considered to form a non‐bonded contact if any two nonhydrogen atoms from different residues are within 4.5 Å. Among these contacts, the interaction between *C*‐terminal tail residue D4829 and HERC2 *C*‐lobe residue R4728 was predominant with a fraction of the population being greater than 0.5 (Figure 7d). This suggests that R4728–D4829 interaction could contribute to the increased stability of the HERC2 extended *C*‐lobe compared to the isolated HERC2 *C*‐lobe alone. However, this one transient interaction is unlikely to be the sole contributing factor for the large difference in melting temperatures observed in the CD melting curves. While the precise reason for the increased stability of the HERC2 extended *C*‐lobe compared to the HERC2 *C*‐lobe remains unclear, our results agree with previous studies that indicate negatively charged *C*‐terminal tails provide stability to the proteins, high mobility group box 1 (HMGB1), and cell division cycle 34 (CDC34) (Anggayasti et al. 2020; Belgrano et al. 2013; Kleiger et al. 2009; Spratt and Shaw 2011).

## 3 DISCUSSION AND CONCLUSIONS

By using complementary biophysical approaches (NMR, CD, and computational analysis), we show that the flexible negatively charged *C*‐terminal tail of HERC2 is primarily disordered when isolated and when it is adjoined to the HERC *C*‐lobe. Interestingly, since the HERC2 *C*‐lobe has a *T*_*m*_ = 55°C and the HERC2 extended *C*‐lobe has a *T*_*m*_ = 71°C, we can conclude that the HERC2 negatively charged *C*‐terminal tail contributes to the increased thermal stability of the HERC2 extended *C*‐lobe construct. While the precise functional role of the HERC2 tail remains unknown, it is postulated that the innate flexibility and many potential binding sites of the negatively charged *C*‐terminal tail could contribute to HERC2‐mediated protein recruitment during the DNA damage response. Future studies are needed to more fully characterize HERC2 protein–protein interactions and clarify how the gigantic HECT E3 ligase plays a role in diseases like cancer and Angelman Syndrome.

## 4 MATERIALS AND METHODS

### 4.1 Simulation protocols and analysis

Constructs for the HERC2 *C*‐lobe (residues G4676–A4797), HERC2 extended *C*‐lobe (residues G4676–H4834), and HERC2 *C*‐terminal tail (residues T4793–H4834) were built so that the *N*‐terminal acetyl groups were patched, and the *C*‐termini were charged. Both the HERC2 *C*‐lobe and HERC2 extended *C*‐lobe constructs contain C4788S and C4773S modifications to be consistent with the constructs used in NMR and circular dichroism spectra. AlphaFold Colab (Tunyasuvunakool et al. 2021) was used to predict the structure of the HERC2 *C*‐lobe and HERC2 extended *C*‐lobe constructs. Amber18 (Case et al. 2018) was employed to carry out molecular dynamic simulations for the HERC2 *C*‐lobe, HERC2 extended *C*‐lobe, and HERC2 *C*‐terminal constructs.

The FF14SBonlysc force field (Nguyen et al. 2014) was used combined with GB‐Neck2 (Nguyen et al. 2013) (igb = 8) and mbondi3 radii (Nguyen et al. 2013). Each system was minimized for 2000 steps, 100 steps of steepest descent followed by the conjugate gradient. The heating process contained 3 steps: Step (1) 1 ns heating from 100 to 300 K with 10.0 kcal/(mol Å^2^) constraints on backbone heavy atoms; Step (2) 250 ps relaxation at 300 K with 1.0 kcal/(mol Å^2^) constraints on backbone heavy atoms; and Step (3) 250 ps relaxation at 300 K with 0.1 kcal/(mol Å^2^) constraints on backbone heavy atoms. Each production run was performed for 1 μs at 300 K with a 4 fs timestep and nonpolar solvation contributions were neglected (gbsa = 0) (Nguyen et al. 2014).

The generalized Born (GB) model was chosen over explicit water models because of the disordered and long *C*‐terminal end of HERC2 (~40 residues). GB describes the instantaneous solvent dielectric response while eliminating the time‐consuming step of water reorganization. This is equivalent to smoothing the energy landscape. In addition, due to the lack of viscosity associated with explicit water molecules, simulations may quickly converge or explore more conformational space using this approach. While the explicit representation is essential for water molecules that play a structural role, the AMBER force field in combination with the GB model has been proven to be accurate in folding simulations of proteins with diverse topologies (Nguyen et al. 2014). Stride (Frishman and Argos 1995) implemented in VMD (Humphrey et al. 1996) was used for the secondary structure assignments. The secondary structure occupancy of a particular residue is defined as the fraction of the number of snapshots that the residue is observed in the α‐helical or β‐strand secondary structural state out of the total number of snapshots used for the analysis.

### 4.2 Construction of HERC2 bacterial expression plasmids

The original DNA construct for the human HERC2 extended *C*‐lobe (Uniprot O95714, residues G4676–H4834) was synthesized by ATUM (Newark, CA) (Villalobos et al. 2006; Welch et al. 2009; Welch et al. 2011). All cysteine residues, except for the catalytic cysteine (C4762), were mutated to serine residues to prevent unwanted disulfide bond formation during purification (C4773S, C4788S). Using compatible 5′ *Bam*HI and 3′ *Xho*I restriction sites, the codon optimized gene was subcloned into an ampicillin‐resistant T7‐inducible pGEX 6P‐2 vector (Novagen) with an *N*‐terminal glutathione‐*S*‐transferase (GST) fusion affinity tag followed by a PreScission Protease cleavage site (LEVLFQ/GP). The resulting GST‐HERC2 extended *C*‐lobe vector was used as the template to produce the HERC2 *C*‐lobe (G4676–A4797) and HERC2 tail (T4793–H4834) constructs. The *C*‐lobe and negatively charged *C*‐terminal tail regions were PCR amplified from the GST‐HERC2 extended *C*‐lobe vector and subcloned into the pGEX 6P‐2 vector using compatible 5′ *Bam*HI and 3′ *Xho*I sites. All plasmids (GST‐HERC2 extended *C*‐lobe, GST‐HERC2 *C*‐lobe, and GST‐HERC2 tail) were isolated using a Monarch Plasmid Miniprep kit (New England Biolabs, Ipswich, MA), quantified using a Nanodrop One^C^ UV–Vis spectrophotometer (Thermo‐Fisher, Waltham, MA), and verified by Sanger DNA sequencing (Macrogen, Cambridge, MA).

### 4.3 Protein expression and purification

The GST‐PreScission‐HERC2 expression plasmids were transformed into *E. coli* strain BL21 (DE3) competent cells (Stratagene) and grown at 37°C in Luria‐Bertani media (2 × 1 L) supplemented with 100 μg/mL ampicillin. For NMR analysis, HERC2 proteins were grown in M9 minimal media (2 × 1 L) supplemented with 100 μg/mL ampicillin, 1 g/L ^15^NH_4_Cl, and 2 g/L ^13^C‐glucose. When the cultures reached an OD_600_ of 0.6–0.8, protein expression was induced with 0.5 mM IPTG for 20 h at 16°C. The cells were harvested by centrifugation (6000*g* for 10 min at 4°C) using a Sorvall LYNX 4000 superspeed centrifuge with a Fiberlite F10‐4x1000 LEX Carbon Fiber rotor (Thermo‐Fisher). Each pellet was resuspended in cold wash buffer (20 mM Tris–HCl, 120 mM NaCl, 2 mM EDTA, and 5 mM DTT, pH 7.4) and supplemented with 1:100 ProBlock Gold Bacterial Protease inhibitor cocktail (GoldBio, St. Louis, MO) and 1 mM phenylmethylsulfonyl fluoride (PMSF). Cells were lysed with an Avestin EmulsiFlex‐C5 Homogenizer (Avestin, Ottawa, ON, Canada) and clarified by ultracentrifugation using an Optima L‐80 XP ultracentrifuge with a Ti 70.1 rotor (Beckman‐Coulter) for 40 min at 41,000 rpm at 4°C. The supernatant was syringe‐filtered with a 0.45 μm filter and applied to 10 mL GST affinity chromatography resin. The GST‐HERC2 protein was eluted with elution buffer (1× PBS, 2 mM EDTA, 10 mM glutathione) and 1 mg PreScission protease was added to cleave the *N*‐terminal GST‐fusion tag. The sample was placed in a 6–8 kDa molecular weight cut‐off membrane and immersed in 2 L of dialysis buffer A (20 mM Tris–HCl, 120 mM NaCl, 2 mM EDTA, 1 mM DTT, pH 8) for 4 h at 4°C. The sample was then transferred to 2 L of dialysis buffer B (20 mM Tris–HCl, 120 mM NaCl, 2 mM EDTA, 1 mM DTT, pH 7.4) and dialyzed overnight at 4°C.

The GST‐fusion tag and PreScission protease were separated from the cleaved HERC2 protein by passing the dialyzed sample through the GST affinity chromatography column a second time. Untagged HERC2 protein was collected in the flow‐through and analyzed for purity by SDS‐PAGE. HERC2 was further purified as needed, using a Superdex75 HiLoad 16/600 size exclusion column and/or a HiTrap Q High Performance 5 mL anion exchange column on an ÄKTA FPLC system. For gel filtration, the sample was concentrated to less than 2 mL using an Amicon Ultra‐15 10 K Centrifugal Filter and loaded onto the Superdex75 column. For anion exchange, the dialyzed sample was buffer matched with the low salt buffer and loaded on the HiTrap Q HP column. A step gradient was used to purify the HERC2 protein that involved a low salt buffer and a high salt buffer (20 mM HEPES, 1000 mM NaCl, 1 mM EDTA, 0.5 mM TCEP, pH 7.4). Contaminants were removed by washing with 30% high salt buffer and the HERC2 protein was eluted with 60% high salt buffer. For gel filtration and anion exchange, the elute proteins were collected in 2 mL fractions and analyzed for purity by SDS‐PAGE.

### 4.4 Heteronuclear NMR spectroscopy

The NMR backbone experiments for the uniformly ^13^C/^15^N‐labeled HERC2 *C*‐lobe (residues G4676–A4797) were collected on a Varian Inova 600‐MHz 4‐channel NMR spectrometer at 25°C in the University of Western Ontario Biomolecular Nuclear Magnetic Resonance Facility. All other spectra for the uniformly ^13^C/^15^N‐labeled HERC2 tail and HERC2 extended *C*‐lobe spectrum were collected on a Varian Inova 600 MHz 4‐channel NMR spectrometer equipped with an HCN cryoprobe at 25°C in the Gustaf H. Carlson School of Chemistry and Biochemistry at Clark University. The samples were prepared in a total volume of 600 μL in a 5 mm i.d. NMR tube such that the sample contained 1.5 mM HERC2 protein, 10% D_2_O, 2 mM 4,4‐dimethyl‐4‐silapentane‐1‐sulfonic acid (DSS) set at 0 ppm, and 2 mM imidazole added as an internal pH indicator (Baryshnikova et al. 2008). The backbone for the HERC2 *C*‐lobe and HERC2 *C*‐terminal tail were assigned using standard 2D and 3D experiments including 2D ^1^H‐^15^N‐HSQC (Kay et al. 1992), 3D HNCACB (Grzesiek and Bax 1992a), 3D CBCA(CO)NH (Grzesiek and Bax 1992b), 3D HNCA (Grzesiek and Bax 1992c; Kay et al. 2011; Muhandiram and Kay 1994), 3D HN(CO)CA (Bax and Ikura 1991; Grzesiek and Bax 1992c), 3D HN(CA)CO (Clubb et al. 1992), and 3D HNCO (Grzesiek and Bax 1992c; Kay et al. 2011; Muhandiram and Kay 1994). Side chain assignments were determined using 3D C(CO)NH (Grzesiek et al. 1993), 3D H(CCO)NH (Grzesiek et al. 1993; Montelione et al. 1992), 3D HCCH‐TOCSY (Bax et al. 1990; Olejniczak et al. 1992), and 3D ^1^H‐^15^N and ^1^H‐^13^C‐NOESY experiments (Marion et al. 1989a; Marion et al. 1989b; Zuiderweg and Fesik 1989). All data were processed using NMRPipe and NMRDraw (Delaglio et al. 1995) and the spectra were analyzed and assigned using the RunAbout tool in NMRViewJ (Johnson 2004; Johnson and Blevins 1994).

### 4.5 Circular dichroism spectroscopy

Circular dichroism spectroscopy was performed on the HERC2 *C*‐lobe (residues G4676–A4797) and HERC2 extended *C*‐lobe (residues G4676–H4834) constructs using a JASCO J‐815 CD spectropolarimeter and 1 mm quartz cuvette. Purified proteins were dialyzed into CD buffer (10 mM Na_2_HPO_4_, 30 mM NaCl, pH 7.4). For the CD spectra wavelength scans, 9 μM HERC2 extended *C*‐lobe and 11 μM HERC2 *C*‐lobe were used. For the CD melting curves, 32 μM HERC2 extended *C*‐lobe and 40 μM HERC2 *C*‐lobe were used. Wavelength scans were recorded from 260 to 195 nm and averaged over 8 accumulations at 5°C using 1 nm increments and an averaging time of 1 s. The melting curves were collected at 222 nm over a temperature range of 5–90°C with a slope 1°C/min, data pitch of 0.2°C, response of 4 s, bandwidth of 1 nm, and a sensitivity of 100 mdeg. The wavelength scans were analyzed with the CONTINLL method using the CDPro software (Provencher and Glöckner 1981; Sreerama and Woody 2000).

## AUTHOR CONTRIBUTIONS

**Kelly L. Waters:** Conceptualization; investigation; writing – original draft; writing – review and editing; formal analysis; visualization. **Kayla J. Rich:** Conceptualization; investigation; formal analysis. **Noah D. Schwaegerle:** Conceptualization; investigation; formal analysis. **Tianyi Yang:** Investigation; formal analysis; conceptualization; visualization; software. **Shuanghong Huo:** Conceptualization; investigation; formal analysis; writing – original draft; visualization; software; supervision. **Donald E. Spratt:** Supervision; project administration; funding acquisition; investigation; conceptualization; writing – original draft; writing – review and editing; formal analysis.
